# Clinical factors affecting colonic iodine-131 distribution after radioactive iodine therapy for thyroid cancer

**DOI:** 10.1007/s11604-025-01882-7

**Published:** 2025-09-30

**Authors:** Noriko Takata, Naoto Kawaguchi, Masao Miyagawa, Akihiro Itou, Reia Yamada, Ayaka Takimoto, Teruhito Kido

**Affiliations:** Department of Radiology, Ehime University Hospital, 454, Shitsukawa, Toon, Ehime Japan

**Keywords:** Radioactive iodine therapy, Thyroid hormone withdrawal, Radiation exposure, Recombinant human thyrotropin, Constipation, Thyroid cancer

## Abstract

**Purpose:**

Radioactive iodine therapy (RAIT) is used to treat patients with thyroid cancer at high risk of recurrence or those with distant metastases. Small amounts of iodine-131 (I-131) are excreted in the stool after RAIT. Thyroid hormone withdrawal (THW) before RAIT can cause constipation, increasing radiation exposure to the colon. Although measuring colonic radiation using I-131 dosimetry would be challenging, colonic radiation dose can be estimated using I-131 whole-body scintigraphy post-RAIT. Therefore, we aimed to determine the clinical risk factors, including THW, associated with colonic distribution on I-131 scintigraphy post-RAIT.

**Materials and methods:**

We analyzed 251 patients who received 530 RAITs, categorized into two groups based on the preparation method. We compared the relationship between clinical risk factors (including preparation method) and colonic I-131 distribution 3 d post-RAIT. In addition, we compared the frequency and degree of colonic I-131 distribution between patients who received RAITs with stimulant laxatives and those who received RAITs with osmotic laxatives. Four subgroup analyses were conducted based on the preparation method and defecation frequency.

**Results:**

We performed 253 RAITs (47.7%) using recombinant human thyrotropin, while the remaining 277 RAITs (52.3%) were performed using THW. In the multivariate analysis, THW, higher RAIT dose (≥ 3.7 GBq), and fewer defecation frequencies (≤ 2 times) were significantly associated with a higher frequency of colonic I-131 distribution (*p* = 0.0206, 0.0020, and 0.0006, respectively). Of the patients treated using THW RAITs, which relieved constipation, those treated with RAITs with stimulant laxatives had significantly lower colonic I-131 distribution than did those treated with RAITs with osmotic laxatives (*p* = 0.0378).

**Conclusion:**

THW, high-dose RAIT, and defecation frequency were significantly associated with colonic I-131 distribution. Pre-treatment strategies, such as the use of stimulant laxatives should be considered to reduce colonic radiation exposure.

## Introduction

Radioactive iodine therapy (RAIT) is used to treat patients with thyroid cancer at high risk of recurrence or those with distant metastases [1]. Iodine-131 (I-131) is primarily excreted in the urine; however, small amounts of I-131 are excreted in the stool [2, 3]. A delay in defecation due to constipation can increase radiation exposure to the colon. Therefore, avoiding constipation during RAIT is essential. Thyroid hormone withdrawal (THW) is a well-known cause of constipation during RAIT [4, 5]. Adequate elevation of thyroid-stimulating hormone is needed to perform RAIT effectively. Although THW has long been performed as a preparation method, it can cause temporary symptoms of hypothyroidism, including constipation. I-131 defecation in stool can be delayed owing to constipation under THW.

Recombinant human thyrotropin (rhTSH) is an alternative RAIT preparation method for patients without distant metastases, demonstrating comparable effectiveness to that of RAIT using THW [5]. Unlike THW, using rhTSH can prevent symptoms caused by hypothyroidism. Consequently, patients using rhTSH are less likely to be constipated and might experience lower colonic radiation exposure than do patients using THW. However, no reports have compared the colonic radiation dose based on the preparation method. Although measuring colonic radiation using I-131 dosimetry would be challenging, colonic radiation dose can be estimated using I-131 whole-body scintigraphy (WBS) post-RAIT.

Therefore, we aimed to determine the clinical risk factors, including THW, associated with colonic distribution on I-131 scintigraphy post-RAIT.

## Methods

### Patients

This study was conducted after approval by our institutional review board (No. 2409004). Informed consent was obtained by an opt-out form on the website. This study included 264 patients with thyroid cancer who underwent RAIT in our institution from 2013 to March 2024. For patients who underwent multiple RAIT sessions, all courses of RAIT were investigated. We included 596 RAITs in this study. However, 61 were excluded because scintigraphy was performed on a day other than 3 d post-RAIT. RAITs without scintigraphy were also excluded (n = 5). Finally, 251 patients with 530 RAITs were analyzed. Regarding the preparation method, we started the rhTSH method in 2013 in patients without distant metastases. Patients with distant metastases received RAIT with THW. The diagnosis of distant metastases was primarily based on whole-body computed tomography (CT) scans. F-18-fluorodeoxyglucose (FDG)-positron emission tomography (PET)/CT was also used when available. RAIT using the THW method was performed in patients with lesions for which the possibility of distant metastases could not be ruled out. When the THW method was used, we changed levothyroxine to liothyronine 4 weeks before RAIT, and liothyronine was discontinued 2 weeks before RAIT. Levothyroxine was restarted after RAIT, and the dose was increased every 3 d until returning to the usual dose. When the rhTSH method was used, patients received 0.9 mg rhTSH injections 1 and 2 d before RAIT. Regardless of the preparation method, patients started a low-iodine diet 2 weeks before RAIT and continued until 3 d after RAIT. The administered dose of I-131 for RAIT was determined based on the presence of macroscopic metastases. Patients without metastases received 30–100 mCi per treatment, while those with metastases received 100–150 mCi per treatment. All patients who received RAITs were hospitalized, and I-131 WBS was performed 3 d post-RAIT, and the patients were discharged.

Colonic I-131 distribution was evaluated based on the frontal image of I-131 WBS 3 d post-RAIT. Colonic distribution was positive if the tracer concentration on the colon was visually higher than that on the mediastinum regardless of the range. The abdominal CT performed to screen metastases was referred to when evaluating colonic distribution. If only gastric uptake was observed, colonic distribution was considered negative. When we analyzed the relationship between preparation method and colonic I-131 distribution, we categorized the colonic distribution patterns into three groups based on the range. The body was divided into right and left sides from the centerline, and we determined if the range of colonic distribution was beyond the centerline. Colonic distribution was classified as follows: 'none' when absent, 'partial' when limited to the right or left side, and 'diffuse' when extending beyond the centerline. Furthermore, the ‘partial’ group was subdivided into two groups based on the side of colonic I-131 distribution. Two radiologists judged the range of colonic distribution in the frontal image of I-131 WBS. All I-131 WBSs were performed using a dual-detector gamma-camera (Infinia; GE Healthcare).

We also analyzed the reproducibility of the colonic I-131 distribution in 160 patients who received RAIT twice or more times. Reproducibility of I-131 distribution was defined as positive when colonic I-131 distributions were observed in all courses of RAITs. For example, in the case of a patient who received RAIT five times, if colonic I-131 distribution was observed in four courses of RAITs, the patient’s reproducibility of colonic distribution was considered negative.

Finally, we analyzed the effect of using laxatives on colonic I-131 distribution. We analyzed 167 RAITs in which only osmotic laxatives (magnesium oxide) or only stimulant laxatives (sodium picosulfate and senna) were used. We compared the frequency and degree of colonic I-131 distribution between two types of laxatives. In addition, to identify the group that effectively reduced colonic I-131 distribution using laxatives, the 167 RAITs were divided into four subgroups based on preparation method and defecation frequency (if defecation occurred thrice or more). The subgroups were: (1) rhTSH RAITs, which relieved constipation, (2) rhTSH RAITs, which did not relieve constipation, (3) THW RAITs, which relieved constipation and (4) THW RAITs, which did not relieve constipation. We also compared the degree of colonic I-131 distribution between the two types of laxatives in each subgroup.

### Statistical analysis

The distribution of continuous variables is summarized as the median (interquartile range). RAITs were divided into two groups based on the preparation method, and we investigated the relationship between colonic distribution on I-131 scintigraphy and preparation method. We also compared the colonic distribution of I-131 scintigraphy and clinical risk factors for RAIT. Clinical risk factors were collected from patient’s medical records and included defecation frequency from the day of RAIT to the day of discharge, age, sex, distant metastases, body mass index, the use of laxatives, and renal function. We analyzed the relationship between clinical risk factors and preparation methods using Pearson’s chi-square test. We also analyzed the relationship between clinical risk factors and colonic I-131 distribution using Pearson’s chi-square test in the univariate analysis, and odds ratios were calculated based on univariate logistic regression analyses. In addition, we performed multivariate analysis using logistic regression analysis. We analyzed the relationship between clinical risk factors and defecation frequency using the Wilcoxon rank sum test. The degree of colonic I-131 distribution between two groups was compared using *p*-value calculated by the chi-square test of 2 × 4 tables. Laterality (left vs. right) within each preparation method was analyzed using the McNemar test (with continuity correction) and confirmed with the exact binomial test. To compare the distribution of ‘Left only’ vs. ‘Right only’ between preparation groups, a Fisher’s exact test (with chi-square test as reference) was performed. A *p*-value of < 0.05 was considered statistically significant. Statistical analyses were performed using JMP software (version 12.0; SAS Institute Inc., Cary, NC, USA).

## Results

In total, 253 (47.7%) RAITs were performed using rhTSH, whereas 277 (52.3%) were performed using THW. Table 1 shows the characteristics of the two RAIT groups based on the preparation method. Table 2 shows univariate and multivariate analyses of clinical factors influencing colonic distribution on I-131 scintigraphy. Regarding the preparation method, positive colonic I-131 distribution was observed in 130/253 (51.4%) RAITs with rhTSH and 217/277 (78.3%) RAITs with THW (*p* < 0.0001). In this analysis, RAIT dose and defecation frequency were identified as significant independent variables in both univariate and multivariate analyses. The presence of distant metastases was significant in univariate analysis but not in multivariate analysis. Table 3 shows the difference in the degree of colonic distribution between the two preparation methods. We analyzed laterality within the preparation methods. In RAITs using rhTSH, the rate of left-only I-131 distribution (23/29 79.3%) was significantly higher than that of right-only distribution (20.7%) (*p* = 0.0030 and 0.0023, by McNemar and exact binomial tests, respectively). In contrast, in RAITs using THW, no significant difference was observed between left-only (24/39 61.5%) and right-only (38.5%) distributions (*p* = 0.200 for both tests). When comparing ‘Left only’ vs. ‘Right only’ distributions between preparation groups, no significant difference was found. Of the 376 RAITs performed with exactly 3.7 GBq, positive colonic I-131 distribution was observed in 75/121 (62.0%) RAITs with rhTSH and 197/255 (77.3%) RAITs with THW (*p* = 0.0020).

**Table 1:** Relationship of RAIT characteristics with the two preparation methods

Parameters	Total (N = 530)	rhTSH method (N = 253)	THW method (N = 277)	*p*-value
Age (years)				
≥ 65	259 (48.9%)	95 (37.5%)	164 (59.2%)	** < 0.0001**
Sex				
Female/Male	329/231 (62.1%)	161/92 (63.6%)	168/109 (60.6%)	0.4791
Distant Metastases				
Yes	228 (43.0%)	0 (0%)	228 (82.3%)	** < 0.0001**
RAIT Dose (GBq)				
≥ 3.7	386 (72.8%)	122 (48.2%)	264 (95.3%)	** < 0.0001**
Defecation Frequency (times)				
≤ 2	260 (49.1%)	119 (47.0%)	141 (50.9%)	0.3737
Using Laxatives				
Yes	225 (42.5%)	92 (36.3%)	133 (48.0%)	**0.0067**
BMI				
≥ 25	185 (34.9%)	82 (32.4%)	103 (37.2%)	0.2496
eGFR (mL/min/1.73m^2^)				
< 60	245 (46.2%)	60 (23.7%)	185 (66.8%)	** < 0.0001**

RAIT: radioactive iodine therapy, rhTSH: recombinant human thyrotropin, THW: thyroid hormone withdrawal, BMI: body mass index, eGFR: estimated glomerular filtration rate

**Table 2:** Univariate and multivariate analyses of clinical factors influencing colonic distribution on I-131 scintigraphy

	Univariate			Multivariate		
Variable	*p*-value	Odds ratio	95% CI	*p*-value	Odds ratio	95% CI
Age (≥ 65 years)	**0.0367**	1.468	1.023–2.106	0.6488	1.102	0.724–1.675
Sex (Female)	0.4981	1.135	0.786–1.640			
Distant Metastases (Yes)	** < 0.0001**	2.802	1.902–4.127	0.7879	0.898	0.392–1.915
RAIT Dose (≥ 3.7 GBq)	** < 0.0001**	3.296	2.214–4.906	**0.0020**	2.103	1.311–3.385
Preparation Method (THW)	** < 0.0001**	3.421	2.346–4.991	**0.0206**	2.472	1.144–5.740
Defecation Frequency (≤ 2 times)	**0.0003**	1.954	1.356–2.815	**0.0006**	1.962	1.335–2.900
Using Laxatives (Yes)	**0.0144**	1.609	1.112–2.330	0.1054	1.397	0.932–2.103
BMI (≥ 25)	0.8665	1.033	0.709–1.505			
eGFR (< 60 mL/min/1.73m^2^)	**0.0013**	1.824	1.263–2.632	0.8613	1.041	0.660–1.634

CI: confidence interval, RAIT: radioactive iodine therapy, THW: thyroid hormone withdrawal, BMI: body mass index, eGFR: estimated glomerular filtration rate

**Table 3:** Differences in the degree of colonic I-131 distribution between the two preparation methods

	Degree of colonic distribution					
Preparation Method	None	Partial (L)	Partial (R)	Diffuse	Total	*p*-value
RAITs using rhTSH	123 (48.6%)	23 (9.1%)	6 (2.4%)	101 (39.9%)	253 (100%)	** < 0.0001**
RAITs using THW	60 (21.7%)	24 (8.7%)	15 (5.4%)	178 (64.3%)	277 (100%)	
Total	183 (34.5%)	47 (8.9%)	21 (4.0%)	279 (52.6%)	530 (100%)	

RAIT: radioactive iodine therapy, rhTSH: recombinant human thyrotropin, THW: thyroid hormone withdrawal, Partial (L): partial (left colon), Partial (R): partial (right colon)

**Fig. 1:**
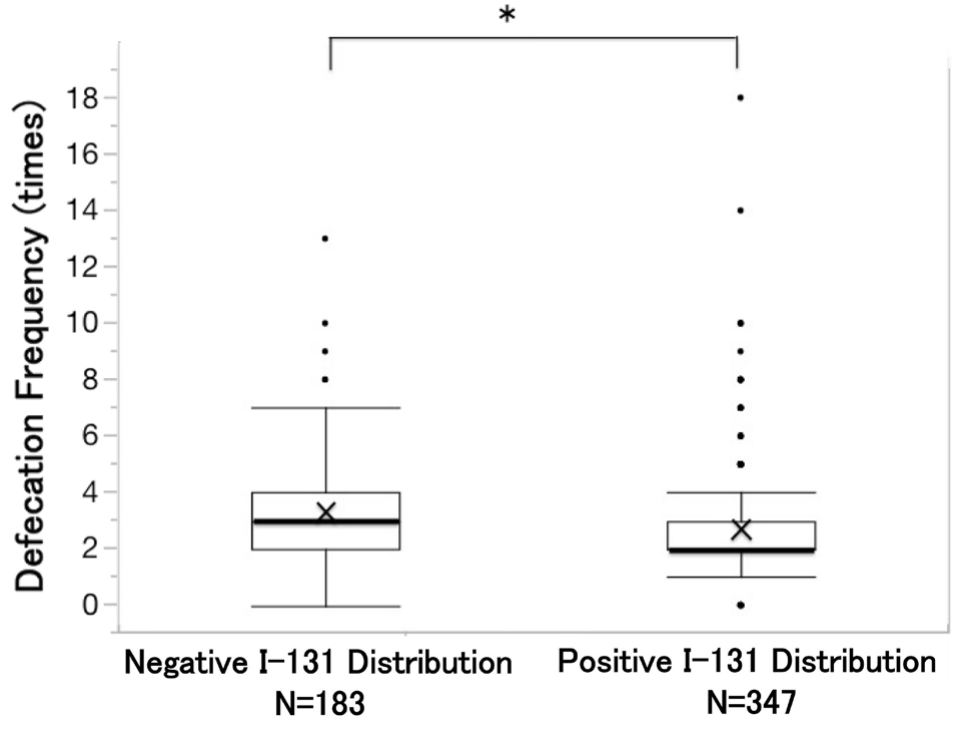
Difference in defecation frequency between negative or positive colonic I-131 distribution groups. Defecation frequency in patients who received radioactive iodine therapy (RAITs) with positive I-131 distribution was significantly higher than that in RAITs with negative I-131 distribution (* *p* = 0.0001)

We also analyzed the relationship between clinical risk factors and defecation frequency from the day of RAIT to the day of discharge. The median defecation frequency was 2.0 [2.0–3.0] in RAITs with colonic I-131 distribution; this was significantly lower than that of RAITs without colonic I-131 distribution (median, 3.0 [2.0–4.0]) (*p* = 0.0001) (Fig. 1). In contrast, no significant difference was observed in defecation frequency between patients who received RAITs using THW and those who received RAITs using rhTSH (*p* = 0.2741). Similarly, no significant difference was observed in defecation frequency between patients who received RAITs with laxatives and those who received RAITs without laxatives (*p* = 0.2136). Among RAITs with laxatives (225 RAITs), laxatives were prescribed after hospitalization in 142 (68.7%) courses of RAITs, whereas in 83 (31.3%) courses of RAITs, laxatives were used regularly before hospitalization. We also investigated the relationship between colonic I-131 distribution and the type of laxative. No difference was observed in defecation frequency between patients who received RAITs with stimulant laxatives and those who received RAITs with osmotic laxatives (*p* = 0.2017).

Of the 160 patients who underwent RAIT treatments, twice or more, 126 patients showed colonic I-131 distribution at one course or more during their treatments. Of these 126 patients, 68 (54%) exhibited colonic I-131 distribution in all treatments, whereas 34 patients did not exhibit colonic I-131 distribution in any of the treatments.

**Fig. 2:**
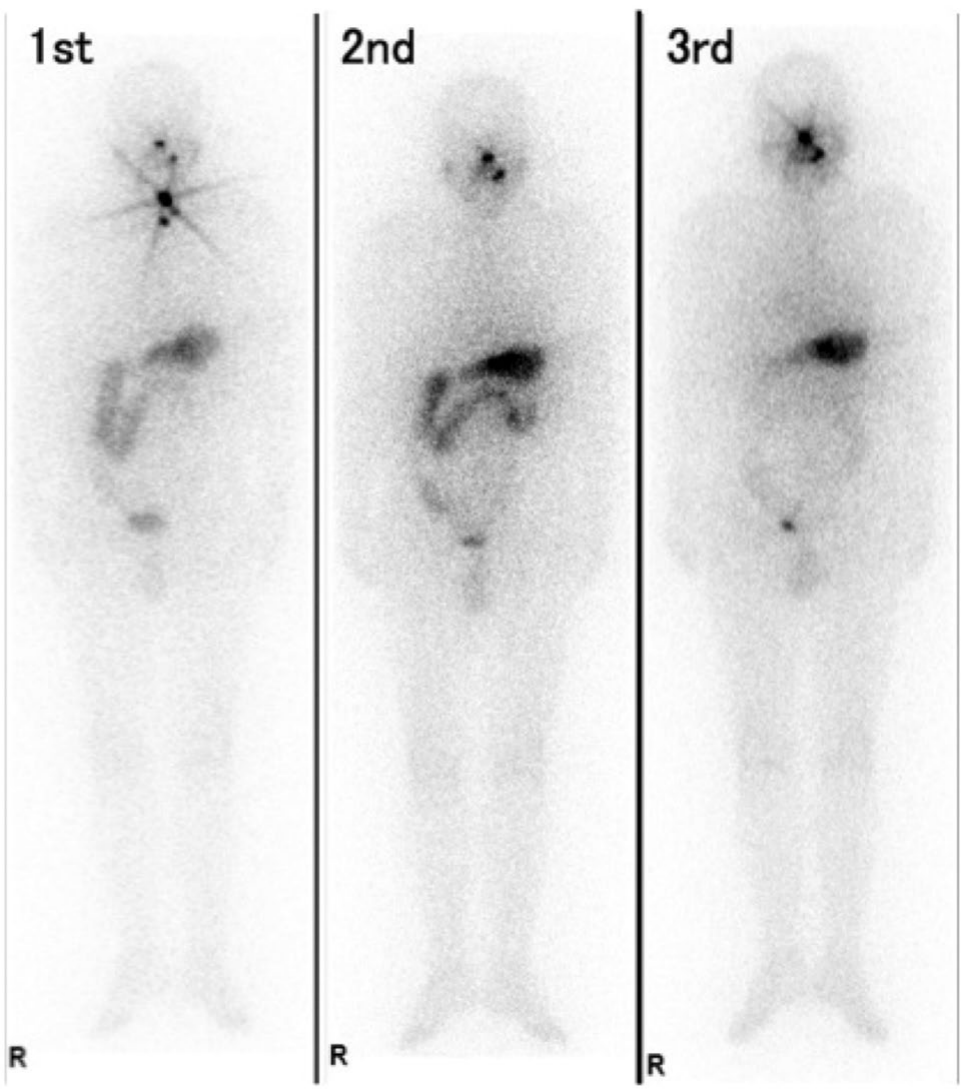
Frontal images of I-131 whole-body scintigraphy after radioactive iodine therapy in a man in his 60 s with lung metastases who received radioactive iodine therapy three times using thyroid hormone withdrawal method. The patient received 1.1 GBq of I-131 in the first RAIT, followed by 3.7 GBq of I-131 at the second and the third RAITs. He received the first and the second RAITs without laxatives. In his third RAIT, as he complained of constipation, he started using 24 mg of senna (stimulant laxative) 1 d before RAIT. As he did not receive quick relief from constipation, he continued taking senna for 3 d. Subsequently, he defecated thrice after RAIT. Colonic I-131 distribution was observed after the first two RAITs; in contrast, in the third RAIT, colonic I-131 distribution disappeared

Finally, we compared the frequency and degree of colonic I-131 distribution between patients who received stimulant and osmotic laxatives. Of the 223 RAITs performed with oral laxatives, 92 patients received RAITs (41.3%) with only stimulant laxatives, 75 patients (33.6%) received RAITs with only osmotic laxatives, and 56 (25.1%) received RAITs with both types of laxatives. The frequency of colonic I-131 distribution showed no difference between patients who received RAITs with only stimulant laxatives and those who received RAITs with only osmotic laxatives, at 62.0% and 70.7%, respectively (*p* = 0.2377). Furthermore, we compared the four subgroups based on the preparation method and condition of constipation. In the subgroup using THW RAITs and in which constipation was relieved (defecating thrice or more), the degree of colonic I-131 distribution with stimulant laxatives was significantly lower than that with osmotic laxatives (Table 4). Figure 2 shows frontal images of I-131 WBS of a patient in this subgroup (his third RAIT).

**Table 4:** Colonic I-131 distribution between two laxative types that relieved constipation in patients receiving THW RAITs

	Degree of colonic distribution					
Type of Laxative	None	Partial (L)	Partial (R)	Diffuse	Total	*p*-value
RAITs with Osmotic Laxative	4 (20.0%)	1 (5.0%)	0 (0.0%)	15 (75.0%)	20 (100%)	**0.0378**
RAITs with Stimulant Laxative	11 (45.8%)	2 (8.3%)	3 (12.5%)	8 (33.3%)	24 (100%)	
Total	15 (34.1%)	3 (6.8%)	3 (6.8%)	23 (52.3%)	44 (100%)	

THW: thyroid hormone withdrawal, RAIT: radioactive iodine therapy, Partial (L): partial (left colon), Partial (R): partial (right colon)

## Discussion

The present study revealed two important findings. First, the frequency of colonic distribution with RAITs using the THW method was significantly higher than that with RAITs using rhTSH. Second, defecation frequency and RAIT dose were significantly associated with the frequency of colonic I-131 distribution. THW can cause constipation during RAIT; however, few reports exist on colonic I-131 distribution post-RAIT. Therefore, this is the first report showing that THW might cause delayed I-131 excretion. Regarding scintigraphy for constipation, reports examining scintigraphy have evaluated colonic transit using oral tracers such as In-111-diethylenetriaminepentaacetic acid [6, 7]. However, such imaging tests are not routine in clinical settings. Its use has not been discussed in I-131 scintigraphy except a report that discusses colonic distribution using I-131-cellulose [8]. Our findings not only showed the cause of increased colonic distribution but also offered a potential strategy to reduce colonic radiation exposure during RAIT.

Our findings showed that defecation frequency was significantly associated with the frequency of colonic I-131 distribution. Regarding age, one-way analysis showed that the frequency of colonic I-131 distribution was significantly higher in older adults than in the young. Age is a risk factor for constipation [9, 10]. Therefore, our findings were consistent with previous reports. However, no significant differences were found in the multivariate analysis. In contrast, other than defecation frequency, preparation method (THW or rhTSH) and I-131 dose were adopted as significant independent variables associated with colonic distribution frequency. Regarding the presence of distant metastases, none of the patients had known involvement of the digestive organs, and no mechanical obstruction of the small or large intestine was observed. We suspect, however, that decreased physical activity due to distant metastases may have contributed to reduced intestinal peristalsis. Since patients with macroscopic distant metastases always received RAITs using the THW method, it was difficult to distinguish the impact of distant metastases from that of the preparation method. Nevertheless, in the multivariate analyses, only the preparation method remained significant, whereas distant metastases did not. This suggests that the effect of the preparation method on colonic I-131 distribution was greater than that of distant metastases. Regarding the RAIT dose, the higher the radiation dose, the greater the amount of unabsorbed tracer. When analysis was limited to RAITs with ‘Left only’ and ‘Right only’ distribution, a predominance of left-sided distribution was observed in RAITs using rhTSH, whereas no laterality was found in RAITs using THW. Since I-131 scintigraphy was performed 3 d post-RAIT, left-sided retention may be more frequently observed at this time point. Although no inter-group difference in laterality was confirmed, the influence of preparation methods on colonic motility might differ between the left and right colon. As previously reported, the left and right colon may be regarded as distinct organs [11]. Thus, our findings may provide a basis for this perspective, and further studies are warranted to clarify these questions.

Colonic I-131 distribution indicates prolonged radiation exposure to the colon. Increased radiation exposure may cause radiation colonitis and exacerbation of inflammation in patients with inflammatory bowel disease. Similar concerns exist for other radionuclide therapies, such as Ra-223, an alpha-emitter used for treating patients with castration-resistant prostate cancer [12], which is excreted in stool. Because of the risk of deterioration of inflammation, careful administration of Ra-223 is recommended in patients with inflammatory bowel disease [13]. In addition, patients with serious constipation are also concerned about relatively higher radiation doses [13]. Likewise, for I-131, which is a beta-emitter, radiation exposure to the colon should not be ignored, and managing constipation may be important when patients with such problems require RAIT.

When the colonic I-131 distribution is observed, the ovaries, as well as the colon, should not be overlooked. Thyroid cancer is more common in women than men [14]. Hence, sex disparities should be considered. High-dose RAIT is not generally associated with female fertility or genetic risks in offspring [15, 16]. However, some reports have described gonadal issues caused by RAIT [17, 18]. High-dose RAIT can decrease the birth rate in women in their late 30’s [17, 18]. Meanwhile, a recent retrospective study [19] reported that a cumulative dose of I-131 > 4.44 GBq did not further increase the risk of infertility, and infertility risk in reproductive-age patients with thyroid cancer was significantly higher than that in the control group, regardless of RAIT. Tumor-related hyper-metabolism adversely affected fertility [19]. The present study evaluated colonic I-131 distribution based on a whole-body planar image only; the radiation dose to the colon and ovaries could not be quantitatively evaluated. According to the International Commission on Radiological Protection-128 publication, the absorbed doses in the upper and lower large intestines are 0.35 mGy/MBq and 0.34 mGy/MBq, respectively [2]. When the patient received 3.7 GBq of RAIT, the absorbed doses of the upper and lower large intestine were calculated as 1.30 Gy and 1.26 Gy, respectively. β-particles from I-131 do not penetrate > 2 mm in depth. Hence, the absorbed dose of ovaries seems not to be serious as long as the colon is distant from the ovaries [20]. The absorbed dose received by each ovary was reported as 140 mGy/3.7 GBq under euthyroid status [16, 18]. However, hypothyroidism under THW causes the reduction of I-131 clearance and results in more prolonged gonadal exposure [18]. Besides, when the colon is close to the ovaries, radiation exposure to the ovaries may be higher than expected. Patients who receive high doses of RAIT should drink plenty of fluids and avoid constipation to minimize radiation exposure to the ovaries [18]. The importance of avoiding constipation during RAIT should be recognized.

Constipation can be relieved to reduce radiation exposure to the colon using laxatives. Notably, the frequency of colonic I-131 distribution in patients who received RAITs with laxatives was significantly higher than that in patients who received RAITs without laxatives, suggesting that the frequency of colonic distribution in patients with constipation might be higher than that in patients without constipation. Among the patients who received RAITs with laxatives (225 RAITs), the laxatives were used after hospitalization in over 50% of the RAITs (142, 68.7%). However, this method could not prevent colonic I-131 distribution. The initiation of laxatives after hospitalization appears to be inadequate, indicating the need for preemptive constipation management. In addition, the question of what kinds of laxatives are effective in reducing colonic radiation exposure remains. Gastrointestinal transit time is longer due to hypothyroidism [21]. We believe that stimulant laxatives, such as sodium picosulfate and senna, may be effective for RAITs using THW. The degree of colonic I-131 distribution in RAITs with stimulant laxatives was significantly lower than that in those with osmotic laxatives in the group using THW RAITs in whom constipation was relieved (Table 4). Therefore, stimulant laxatives may be more effective in reducing colonic I-131 distribution after RAIT.

This study has some limitations. First, this was a retrospective study, and we could not obtain adequate information on stool volume and hardness from patients’ medical records; we thus had no choice but to rely on defecation frequency alone. Moreover, we were unable to include baseline information, such as bowel movement frequency and medication history, due to the retrospective design of this study. A more precise evaluation of the effect of laxatives on colonic I-131 distribution would require analysis incorporating these factors that may affect bowel motility at the time of RAIT. Further prospective studies are warranted to address this issue. Second, we performed only qualitative analyses of colonic I-131 distribution, not quantitative ones. The exact absorbed dose to the colon remains unclear; however, unnecessary radiation exposure should be minimized. Further prospective studies will be needed to reveal whether colonic radiation dose during RAIT is associated with acute and late toxicities to the colon in future. Finally, the diagnosis of distant metastases was not based on pathologic confirmation but on imaging findings. Among the 49 RAITs (17.7%) using the THW methods, patients had lesions that could not be definitely diagnosed as distant metastases. For example, solitary pulmonary nodules and granular opacities—most likely inflammatory in nature—were included. In this study, RAIT cases with such lesions were classified as having no distant metastases. These classifications may not fully reflect the actual disease status.

## Conclusions

THW, high-dose RAIT, and defecation frequency were significantly associated with colonic I-131 distribution post-RAIT. Pre-treatment strategies, such as the use of stimulant laxatives, are necessary to reduce colonic radiation exposure.
